# Time Course and Determinants of Individual Motivation among Women Enrolled in a Diet and Physical Activity Primary Prevention Trial

**DOI:** 10.3390/ijerph17228589

**Published:** 2020-11-19

**Authors:** Saverio Caini, Melania Assedi, Elisa Grechi, Ilaria Ermini, Donatella Zagni, Daniela Occhini, Maria Castaldo, Benedetta Bendinelli, Domenico Palli, Giovanna Masala

**Affiliations:** 1 Cancer Risk Factors and Life-Style Epidemiology Unit, Institute for Cancer Research, Prevention and Clinical Network (ISPRO), 50139 Florence, Italy; s.caini@ispro.toscana.it (S.C.); m.assedi@ispro.toscana.it (M.A.); i.ermini@ispro.toscana.it (I.E.); d.zagni@ispro.toscana.it (D.Z.); danielaocchini@yahoo.it (D.O.); maria_castaldo@hotmail.it (M.C.); b.bendinelli@ispro.toscana.it (B.B.); d.palli@ispro.toscana.it (D.P.); 2 The Italian League Against Tumors (LILT), Oncological Rehabilitation Centre (CeRiOn), 50139 Florence, Italy; elisa.grechi@ipsico.org

**Keywords:** motivation, dietary habits, physical activity levels, primary prevention, randomized intervention trial

## Abstract

We studied the determinants of motivation among post-menopausal women enrolled in a two-year diet and physical activity primary prevention randomized trial. Participants were requested to grade the importance attached to changing their lifestyle, their confidence about being able to implement the change, and their willingness to be involved in studies focusing on lifestyle. We used multi-adjusted regression to investigate the association between individual characteristics, study arm, and individual motivation at study entry and end. Participants (*n* = 234) were highly motivated both at entry and throughout the study. Women with pre-existing healthier eating habits and lifestyles (e.g., high consumption of fruit and vegetables, low red meat consumption, and physically active) were more motivated at entry and over the course of the study. Women assigned to any intervention arm were more motivated than those in the control arm. These findings may help enhance adherence to recommendations and improve effectiveness of community-based health promotion campaigns.

## 1. Introduction

There is now solid and unquestionable scientific evidence that several lifestyle characteristics, such as unhealthy dietary habits (e.g., intake of red and processed meat or high glycemic index), tobacco smoking, alcohol abuse, and lack of physical activity, as well as inability to effectively cope with stress, increase the risk of chronic degenerative diseases, such as cancer at several body sites, cardiovascular disease, stroke, type 2 diabetes, osteoporosis, and obesity. Of note, many of those risk factors tend to co-occur, often interacting in ways that amplify their negative effects on health, and are associated with an increase in risk of a wide range of conditions (such as overweight/obesity, hypertension, or hypercholesterolemia) and chronic diseases, which may negatively impact the course of each other and, eventually, people’s length and quality of life. Based on these considerations, there is widespread agreement on the need to promote the adoption of healthy lifestyles in order to reduce the burden of disease attributable to these pathologies [1]. However, it is usually very challenging to persuade people to change their lifestyle (though harmful to health) as this is determined by a complex range of social pressures and unconscious psychological mechanisms that may be exceedingly hard to escape [2,3].

Several trials have investigated the efficacy of face-to-face or group counselling as a tool to achieve health-related objectives, such as changes in dietary habits and physical activity levels [3,4,5,6,7], regular breast self-examination [8], or glycemic control in patients with diabetes [9]. Much less attention has been given by researchers to the personal experiences and perspectives of individuals enrolled in primary prevention trials and requested to modify their habits or behaviors [10,11,12]. However, the maintenance of a positive attitude towards the requested change and the enhancement of individual motivation are likely to be important determinants of adherence to the proposed intervention and, ultimately, a requirement for its effectiveness at a population level.

The diet, physical activity and mammography (DAMA) study is a two-year primary prevention randomized intervention trial (Trial registration ID: ISRCTN28492718) with a 2 × 2 factorial design that aims to investigate whether mammographic breast density (MBD) can be reduced among post-menopausal women by modifying their dietary habits and/or physical activity levels [13]. Women assigned to any of the three intervention arms were requested to modify their dietary habits and/or physical activity levels and maintain this change for the whole duration of the study. During the trial, participants’ motivation was monitored by means of specifically developed questionnaires that were administered at the beginning, middle, and end of the study. The results related to the primary outcome of the study have been published recently, showing that women in the diet and in the physical activity intervention arms (but not those in the double intervention arm) had a reduction in their mammographic breast density compared to the control group [14]. Here, we describe how participants’ motivation changed over time and investigate which factors were associated with individual motivation at baseline and at the study’s end.

## 2. Materials and Methods

### 2.1. The DAMA (Diet, Physical Activity and Mammography) Study

The rationale and methodology of the DAMA study have been described in detail elsewhere [13]. In brief, study participants were menopausal women aged 50 to 69 years, with high mammographic breast density at routine screening according to the Breast Imaging-Reporting and Data System (BI-RADS) classification (category 3 or 4, i.e., mammographic breast density > 50%), non-smokers, not taking hormone replacement therapy, and with no major co-morbidities (such as cancer, diabetes, or cardiovascular diseases) at enrolment. Women that were called for a second-stage diagnostic procedure following the screening mammography were considered as not eligible for the study, regardless of whether they were eventually diagnosed with breast cancer.

Prior to randomization, each participating woman was requested to have resting blood pressure, heart rate, weight, height, and waist and hip circumferences measured by the study personnel and to provide fasting venous blood and urine samples, which were aliquoted and stored in a dedicated biobank. Information on dietary habits and lifestyle (including physical activity levels) was collected by means of two self-administered questionnaires, previously used in the frame of the European Prospective Investigation into Cancer and Nutrition (EPIC) study [15]. In detail, the EPIC study’s food frequency questionnaire was specifically validated for Italian dietary habits and allowed to collect data on the frequency of consumption, and the quantity consumed, of a large variety of food items. The lifestyle questionnaire covered demographics, smoking habits, consumption of alcoholic beverages, medical history, menstrual and reproductive history, and use of exogenous sex hormones and included a section with questions on physical activity at work, at home, and during leisure time. All of the above procedures were repeated once more at the end of the two-year study.

Each woman was then assigned to one of the four study arms through permuted block randomization (*n* = 4) stratified by age (50–59 or 60–69 years) and body mass index (body mass index; <25 or ≥25 kg/m^2^). Women assigned to the dietary intervention (group 1) were requested to adopt a diet based on plant foods (cereals, vegetables, legumes, and fruits), with a low glycemic load and low in alcohol and saturated and trans fats, and to attend periodic small-group meetings and cooking classes. Women assigned to the physical activity intervention (group 2) were requested to gradually increase their recreational physical activity levels in order to reach a daily energy expenditure of 3 MET-hours (MET = metabolic equivalent) [16], and to attend a 1-h exercise session (led by a professional trainer) per week and periodic small-group meetings and collective walks. Women in the dietary + physical activity arm (group 3) were requested to adhere to both intervention protocols. Finally, women assigned to the control arm (group 4) were given general advice on healthy lifestyle for the prevention of cancer and invited to attend one group meeting at the beginning of the study.

Adherence to the intervention was periodically monitored by means of diet and physical activity diaries and 24-h recalls. In detail, study participants assigned to any intervention arm were requested to keep five written 1-week diaries focusing on diet (group 1), physical activity (group 2), or both (group 3) over the two-year study period. The diaries were reviewed by the study personnel (dieticians and exercise experts) and discussed with the participants during individual phone calls in order to agree on a plan to overcome any difficulties encountered in trying to achieve all the objectives of the study. In addition, four 24-h recalls on diet and physical activity (one in each season of the year) were administered to all participants of the study, including those in the control group.

### 2.2. The Motivation Questionnaire

The motivation questionnaire of the DAMA study was developed following the theory and methods by Rollnick et al. [17], which were based on the methodology of motivational interviewing developed by Miller and on the Prochaska and DiClemente’s “stages of change” model [18,19]. According to the latter, people constantly change their willingness to engage in a process of behavior change. Individual willingness varies along a continuum and is determined by a number of intertwined factors, the most important of which are importance and confidence. Importance (“Why would I make this change? Is it worth?”) is the value that a person attributes to the change, which critically depends on the expectations about the consequences that the change entails. Confidence (“Would I be able to?”) refers to a personal sense of self-efficacy, i.e., one’s ability to make and maintain the requested (and/or desired) change.

Consistent with this approach, the key concepts that we aimed to measure were the importance that each participating woman attached to a change in her lifestyle (dietary habits and physical activity levels), the confidence about her ability to implement the change, and the willingness to be involved in research studies focusing on individual lifestyle. In detail, the motivation questionnaire of the DAMA study included five Likert-scale questions:

- How much do you think it is important to change your eating habits?
- How much do you think it is important to change your physical activity levels?
- How confident do you feel of being able to change your eating habits?
- How confident do you feel of being able to change your physical activity levels?
- How willing do you feel to participate in this type of research?

The motivation questionnaire was administered to all participants at baseline (i.e., prior to randomization), midway, and at the end of the study, except that midway through the study, women in the diet intervention arm were not requested to answer the questions on physical activity, and those in the physical activity intervention arm were not asked the question on dietary habits, in order to contain the risk of contamination. At each administration of the questionnaire, women were requested to respond to each question by assigning a score between 0 (“not at all”) and 10 (“totally”).

### 2.3. Statistical Analysis

We calculated the mean (with standard deviation, SD) and median (with quantile deviation, QD) scores to each question at baseline, midway, and end-of-study, overall and by study arm.

We fitted multiple logistic regression models to investigate factors associated with the odds of being in the upper vs. lower half of the distribution of the scores to each question in the baseline motivation questionnaire. Factors that were taken into consideration included age at enrolment, smoking habits, body mass index, waist circumference, marital status, education level, consumption of specific foods and food groups (vegetables, legumes, fruit, olive oil, red and processed meat, fish and seafood, rice and pasta, potatoes, coffee, and dairy) and alcoholic beverages, and physical activity at home, at work, and in leisure time. Factors that were positively or negatively associated (with a *p*-value ≤ 0.10) with motivation in univariate analysis were included in a multiple logistic regression model; age, smoking habits, and body mass index were included regardless of their *p*-value in a univariate analysis. This procedure was repeated for each of the five questions included in the motivation questionnaire.

We then fitted multiple linear regression models to explore factors associated with the change in motivation (each of the five questions) between the baseline and the end-of-study questionnaire. The procedure adopted to obtain the final model is similar to that described above, except that all models included, as an additional covariate, the corresponding score at the baseline questionnaire. In these models, a beta coefficient significantly above the null value means that the motivation score either increased more, or declined less, between the baseline and the end-of-study questionnaires than among women in the reference group.

All tests were two-sided, with *p*-values < 0.05 considered as statistically significant. All analyses were conducted using STATA version 14 (STATA Corp., College Station, TX, USA).

### 2.4. Ethics

All the procedures performed in this study were in accordance with the ethical standards of the local research committee (Comitato Etico Azienda Sanitaria Firenze, reference number: 15/2007/CEL) and with the declaration of Helsinki of 1975, revised in 2013. Informed consent was obtained from all individual participants included in the study.

## 3. Results

**Figure 1:**
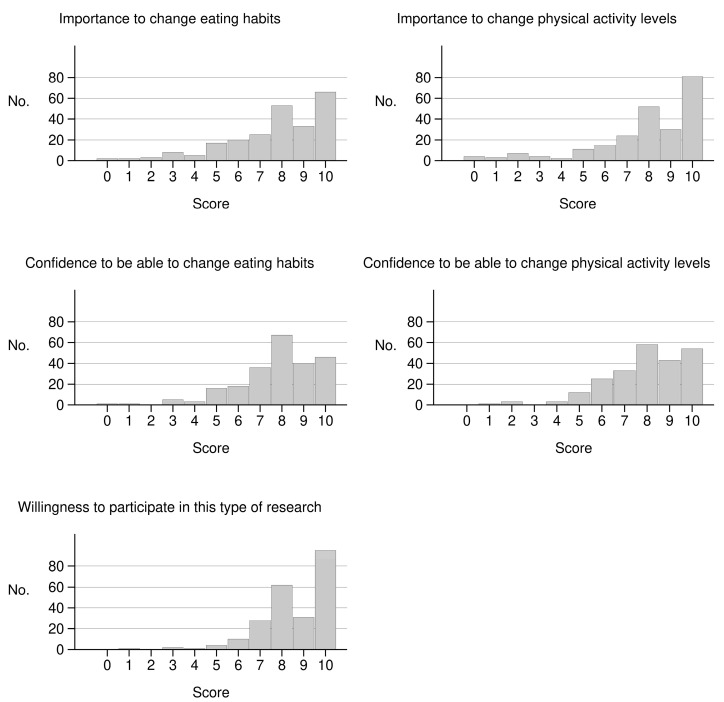
Distribution of responses to the baseline motivation questionnaire in the diet, physical activity and mammography (DAMA) trial. Florence, Italy, 2009–2012.

The distribution of the variables measuring participants’ motivation at the study baseline is shown in Figure 1. The mode was 10 for both questions on importance to change and on willingness to participate and 8 for both questions on confidence of being able to change. The median value was 9 (QD 2) for the question on willingness to participate and 8 (QD 2) for the other questions.

### 3.1. Lifestyle Factors Associated with Motivation at Study Baseline

Table 1 shows the dietary and physical activity variables that were associated (*p* < 0.10) with motivation at the study baseline. In most cases, healthy eating and lifestyle habits were associated with higher motivation, and vice versa. For instance, higher consumption of olive oil and of fruit and increasing levels of household physical activity were associated with higher scores in four, two, and two questions, respectively, of the motivation questionnaire, while higher consumption of red and processed meat was associated with lower scores in three questions. A few exceptions existed, namely the inverse association between increasing levels of recreational physical activity and the importance to change one’s levels of physical activity (odds ratio (OR) 0.33; 95% confidence intervals (CI) 0.15–0.72, *p*-value 0.015) and between higher consumption of vegetables and the confidence of being able to change one’s physical activity levels (OR 0.31; 95% CI 0.10–0.93, *p*-value 0.044).

### 3.2. Changes in Motivation during the Study and Associated Factors

There was a slight increase (≈1 point) between the baseline and end-of-study questionnaires in the mean score to both questions on importance to change one’s lifestyle, which was somehow milder among women in the control group (Figures S1–S5). The mean score to both questions on confidence to change one’s lifestyle did not change between the baseline and end-of-study questionnaire, except for a moderate decrease (<0.5 points) among women in the control group. There were minimal changes (<0.5 points) between the baseline and end-of-study questionnaires in all study arms for the question on willingness to participate. Scores of the baseline and end-of-study questionnaires tended to correlate (Table S1), despite some degree of regression to the mean.

We report, in Table 2, the factors associated with a change in motivation between the baseline and the end of the study. Overall, women in any of the three intervention arms tended to be more motivated at the end of the study than women in the control arm. This was especially evident for women in the dietary study arm (group 1), where the adjusted β coefficient quantifying the mean difference in score between the two time points ranged between +0.66 and +0.81 for the four questions on importance and confidence, always achieving statistical significance. Women in the physical activity study arm had significantly higher scores on the two questions on physical activity at the end-of-study questionnaire, while the scores in the two questions on eating habits increased, despite not achieving statistical significance (β +0.55, *p*-value 0.072 for importance and β +0.049, *p*-value 0.129 for confidence). The scores increased between the study’s baseline and end also increased among women in the double intervention arm (group 4), although reaching statistical significance only for the question on confidence to be able to change one’s eating habits (β +0.67, *p*-value 0.043). The willingness to participate in similar research studies did not significantly vary over time among women in any study arm. Finally, higher consumptions of red and processed meat, rice and pasta, and wine were generally associated with lower motivation at the end of the study, while the opposite was observed for the consumption of dairy and legumes and for the time spent walking.

## 4. Discussion

We investigated which factors were associated with motivation at the study baseline and with its change over the course of the study, among post-menopausal women participating in a primary prevention trial. The study participants appeared to be highly motivated at their first encounter with the study personnel (i.e., before randomization), and their motivation tended to remain fairly high during the two-year intervention. By and large, a pre-existing healthier lifestyle was positively associated with a higher motivation both at baseline and over the course of the study. In addition, women assigned to any intervention arm (i.e., who were requested to change their dietary habits, physical activity levels, or both) had a more favorable course of individual motivation than those assigned to the control group.

The observation that individual motivation was very high at the study baseline came as no surprise and was most likely due to a self-selection process of participants (30.4% of eligible women agreed to join the study) [14]. The study sample consisted of women who had expressed their willingness to participate in a two-year intervention study and modify their dietary habits and/or physical activity levels in accordance with the instructions received. This was a demanding task, so it appears reasonable to assume that the recruitment process selected a group of highly motivated women. The recognition of the importance of a change (in dietary habits and/or physical activity levels) was greater, at baseline, than the confidence to be able to implement the change. This is consistent with the Prochaska and DiClemente transtheoretical model of the stages of change [20], according to which the “contemplation” stage (recognition that a given behavior is problematic) precedes the “preparation” stage (intention to take action). Despite inter-individual differences, it is also worth highlighting how the motivation remained generally high among women assigned to any study arm. By the frequent contact with the study personnel (both in person, e.g., during periodic small-group meetings, cooking classes, and walks, and by telephone, e.g., when administering the 24-h recalls or reviewing the 1-week diaries), participants may have felt constantly cared for and encouraged in their efforts, which may have contributed to keeping their motivation high throughout the study. The finding of consistently high motivation is all the more remarkable when considering that women were not approached because of being “diseased” or “at risk of disease”, nor addressed in this way by the study personnel at any time during the study.

At the study baseline, the women who felt it was most important to change their lifestyle and declared to be most confident to succeed were those who had a comparatively healthier lifestyle: higher consumption of fruit and olive oil, less consumption of meat and pasta, more household physical activity, and lower intake of wine. This phenomenon can be regarded as akin to the healthy user and healthy adherer biases that are sometimes at play in pharmacological treatment trials [21,22] or to the greater propensity of healthier people to engage in cancer screening programs [23]. Selection bias is a common cause of concern in designing lifestyle intervention trials, and although it does not invalidate the conclusions that can be drawn from such studies, it may limit generalizability and applicability of results to a real-world population [24].

Women with a comparatively healthier lifestyle at study baseline also had a more favorable course of motivation throughout the study period. One more factor that positively influenced the evolution of motivation over time was the assignment to any of the intervention arms, which may have different explanations. Women allocated to the control arm of lifestyle intervention studies may sometimes be disappointed and frustrated by of the lack of involvement in the study activities (frequently seen as attractive because of the perceived positive effect on health), and this can lead to a decline in motivation [24]. However, this does not appear to apply here, as the scores in the two questions on importance tended to increase over time among all participants, no matter the study arm to which they were assigned. Alternatively, early success in implementing the requested change may have triggered a positive feedback loop able to increase one’s motivation (or maintain it at high levels) throughout the course of the study in the intervention arms. Beyond the control arm, women assigned to the double intervention arm appeared to experience a less favorable course of motivation than those in the two single-intervention arms, especially for what concerns the questions on physical activity and on the willingness to participate in lifestyle-focused research projects in the future. Fairly consistently, an association between the intervention and MBD (the primary outcome of the DAMA trial) emerged among women assigned to the dietary or physical activity arm only, but not among those in the double intervention arm [14]. While a chance finding cannot be ruled out, it seems plausible to hypothesize that the request for a change of multiple aspects of one’s lifestyle, and the consequent difficulty in achieving the set goals, may have led to a slight loss of motivation, especially in the long term (i.e., at the end of the study). Finally, while the motivational intervention for change according to the trans-theoretical model is applicable to a wide range of different behaviors, only a single behavior at a time is usually targeted, as it is believed that it is much more difficult to effectively negotiate multiple changes in one’s lifestyle at the same time. The observed results for the double intervention study arm seem to confirm this belief.

Willingness to participate in research studies evolved less favorably over time among women requested to change their physical activity levels (alone or in conjunction with dietary habits) than among women who were only requested to change their dietary habits. This may be the consequence of the physical activity intervention being somewhat more demanding than the dietary intervention, as the former included the attendance of weekly exercise sessions led by a professional trainer, while the dietary intervention was self-managed to a much greater extent. Previous research has highlighted that any requested change in one’s lifestyle (either defined by healthcare professionals or self-set) must be commensurate with individual skills, abilities, and training, while setting overly ambitious goals may be counterproductive and preclude success [25,26]. In addition, the promotion of multiple health behavior changes may sometimes be less effective than single interventions [27]. Translatability of research findings into effective field interventions may be hampered by the failure to take into account these aspects.

Readiness to change is a state of mind that results from a complex psychical activity, and we felt it important to understand how this varied over time during the participants’ attempt to change their lifestyle. The concepts of importance and confidence and the methodology adopted for their assessment helped us achieve this objective, allowing us to know the level of motivation of each participant from the very beginning of the study and to monitor it during the course of the study. The transtheoretical model of the stages of change states that people are likely to move between stages in a cyclical manner, and the use of a motivation questionnaire helped us identify (alongside other tools, namely the diaries and 24-h recalls) the needs and difficulties experienced by each participant. The questionnaire represented a structured yet easy-to-use way to help people express what they felt about the requested changes. Moreover, the motivation assessment was centered on the person, as it allowed the respondent to focus on the area that the person herself felt as the most in need of attention. Finally, the concept of confidence is also closely related to that of self-efficacy, as the latter refers to a person’s confidence in the ability to achieve a specific behavioral change. Self-efficacy may vary according to the different situations and requests, and it is important to underline that acting and engaging in a change may be one of the most effective ways to enhance one’s self-efficacy.

The major strengths of our study are the large study size, the use of a multi-dimensional motivation questionnaire that was developed ad hoc for the study, and the availability of data collected at three points in time. Importantly, the first administration of the questionnaire preceded the randomization—this made it possible to adjust for pre-study motivation scores and assess more accurately the determinants of change over time. Our study has several limitations. The lifestyle, dietary, and motivation questionnaires were not administered to eligible women who eventually chose not to participate—this prevented us from assessing the presence of self-selection bias and evaluate its extent. Although the motivation questionnaire was developed based on an established methodology of motivational interviewing [17] by adapting questions to our specific context, no validation of the questionnaire was conducted before administering it to the participants in the DAMA study. Motivation scores regressed to the mean over the course of the study—this is frequently observed in longitudinal studies, particularly in the presence of large intra-individual variability or substantial measurement errors, and failure to take it into account may lead to an erroneous interpretation of study results [28]. Entering baseline values in the models should have largely neutralized this possible source of bias, although possibly not completely. Finally, the study participants might have been influenced, in responding to the motivation questionnaire, by the desire (perhaps unconscious) to meet the expectations of the study personnel, which might have resulted in somewhat inflated scores.

## 5. Conclusions

In conclusion, we found that agreeing to engage in a lifestyle change program has a generally positive effect on one’s motivation, although a major role is also played by pre-intervention individual lifestyle. This finding originates from an experimental study and its translatability into real-world settings is to be verified, yet it can prove valuable when planning large-scale, community-based health promotion campaigns. It may be challenging to achieve the necessary motivation to embark on adopting a healthier lifestyle, especially for those who need it most; once this step is taken, however, a person may enter a virtuous circle of enhanced motivation and self-efficacy, which may ultimately facilitate the achievement of self-set goals. Therefore, it is critical that a person is adequately supported during the early stages of a lifestyle change program, when he/she is likely to be more sensitive to both positive and negative feedback. In this regard, knowing the determinants of individual motivation and their distribution in the population targeted by a public health intervention may allow for identifying population subgroups requiring a differentiated approach, thus possibly enhancing the overall effectiveness of the intervention itself.

## Figures and Tables

**Table 1:** Factors associated with the odds of being in the upper vs. lower half of the distribution of scores given to each question of the baseline motivation questionnaire in the diet, physical activity and mammography trial. Florence, Italy, 2009–2012.

		OR ^(a)^	Lower 95% CI	Upper 95% CI	*p* ^(b)^
How much do you think it is important to change your eating habits?					
Red and processed meat	3rd vs. 1st tertile	0.33	0.15	0.72	0.005
Sweets, biscuits and cakes	3rd vs. 1st tertile	2.02	0.94	4.32	0.085
Olive oil	3rd vs. 1st tertile	3.71	1.74	7.90	0.001
Household physical activity	3rd vs. 1st tertile	2.01	0.96	4.23	0.064
How confident do you feel of being able to change your eating habits?					
Drinks wine	current vs. never	0.40	0.15	1.08	0.071
Fruit	3rd vs. 1st tertile	2.13	0.99	4.57	0.055
Rice and pasta	3rd vs. 1st tertile	0.44	0.21	0.92	0.031
Coffee	3rd vs. 1st tertile	0.33	0.15	0.72	0.005
Household physical activity	3rd vs. 1st tertile	2.04	0.97	4.30	0.058
How much do you think it is important to change your physical activity levels?					
Red and processed meat	3rd vs. 1st tertile	0.46	0.22	0.99	0.049
Olive oil	3rd vs. 1st tertile	2.85	1.31	6.19	0.009
Recreational physical activity	3rd vs. 1st tertile	0.33	0.15	0.72	0.005
How confident do you feel of being able to change your physical activity levels?					
Vegetables	3rd vs. 1st tertile	0.31	0.10	0.93	0.044
Fruit	3rd vs. 1st tertile	2.49	1.12	5.54	0.028
Red and processed meat	3rd vs. 1st tertile	0.47	0.22	1.02	0.056
Olive oil	3rd vs. 1st tertile	4.34	1.44	13.07	0.015
How willing do you feel to participate in this type of research?					
Drinks wine	current vs. never	0.45	0.19	1.05	0.063
Rice and pasta	3rd vs. 1st tertile	0.36	0.18	0.74	0.006
Olive oil	3rd vs. 1st tertile	3.80	1.84	7.87	<0.001
Coffee	3rd vs. 1st tertile	0.49	0.24	0.98	0.043

OR: odds ratio. CI: confidence intervals; ^(a)^ Multiple unconditional logistic regression models adjusted by age, smoking habits, and body mass index; ^(b)^ *p*-value is for trend for variables that were modelled in tertiles. Variables were only shown for which the *p*-value was ≤ 0.10.

**Table 2:** Factors associated with the change in motivation between baseline and end-of-study questionnaires. The diet, physical activity and mammography trial. Florence, Italy, 2009–2012.

		β ^(a)^	Lower 95% CI	Upper 95% CI	*p* ^(b)^
How much do you think it is important to change your eating habits?					
Study arm	Diet	0.81	0.21	1.40	0.008
Study arm	Physical activity	0.55	−0.05	1.15	0.072
Study arm	Diet + physical activity	0.60	−0.01	1.20	0.053
Smoking	Former vs. never	0.45	0.01	0.89	0.044
How confident do you feel of being able to change your eating habits?					
Study arm	Diet	0.66	0.04	1.28	0.038
Study arm	Physical activity	0.49	−0.14	1.12	0.129
Study arm	Diet + physical activity	0.67	0.02	1.31	0.043
Red and processed meat	3rd vs. 1st tertile	−1.00	−1.55	−0.45	<0.001
Fish	3rd vs. 1st tertile	0.52	−0.06	1.10	0.075
Wine	3rd vs. 1st tertile	−0.69	−1.35	−0.03	0.038
Dairy	3rd vs. 1st tertile	0.74	0.19	1.28	0.008
Walking	3rd vs. 1st tertile	0.58	−0.01	1.16	0.053
How much do you think it is important to change your physical activity levels?					
Study arm	Diet	0.71	0.16	1.26	0.012
Study arm	Physical activity	0.84	0.28	1.39	0.003
Study arm	Diet + physical activity	0.54	−0.02	1.11	0.059
Red and processed meat	3rd vs. 1st tertile	−0.48	−0.97	0.01	0.055
Rice and pasta	3rd vs. 1st tertile	−0.62	−1.12	−0.13	0.014
Dairy	3rd vs. 1st tertile	0.50	0.01	1.00	0.042
How confident do you feel of being able to change your physical activity levels?					
Study arm	Diet	0.73	0.08	1.38	0.028
Study arm	Physical activity	0.97	0.31	1.63	0.004
Study arm	Diet + physical activity	0.27	−0.39	0.92	0.428
Red and processed meat	3rd vs. 1st tertile	−0.63	−1.20	−0.07	0.029
Legumes	3rd vs. 1st tertile	0.66	0.07	1.24	0.030
Walking	3rd vs. 1st tertile	1.09	0.50	1.68	<0.001
How willing do you feel to participate in this type of research?					
Study arm	Diet	0.73	−0.09	1.56	0.080
Study arm	Physical activity	0.12	−0.69	0.94	0.767
Study arm	Diet + physical activity	0.17	−0.68	1.01	0.699
Red and processed meat	3rd vs. 1st tertile	−0.85	−1.58	−0.11	0.022
Rice and pasta	3rd vs. 1st tertile	−0.67	−1.39	0.06	0.073
Wine	3rd vs. 1st tertile	−1.49	−2.35	−0.64	0.003

CI: confidence intervals. ^(a)^ Multiple linear regression models adjusted by value at baseline, age, and body mass index; ^(b)^ *p*-value is for the trend for variables that were modelled in tertiles. Variables were only shown for which the *p*-value was ≤ 0.10.

## Supplementary Materials

The following are available online at https://www.mdpi.com/1660-4601/17/22/8589/s1. Table S1. Median (with quantile deviation, QD) and mean (with standard deviation, SD) score in the end-of-study motivation questionnaire by categories of score in the baseline motivation questionnaire. The Diet, Physical Activity and Mammography trial. Florence, Italy, 2009–2012; Figure S1. Mean score to the question about importance to change eating habits at the baseline, midway and end-of-study motivation questionnaire, by study arm (D: diet; PA: physical activity; D+PA: diet and physical activity; C: control). The Diet, Physical Activity and Mammography trial, Florence, Italy, 2009–2012; Figure S2. Mean score to the question about importance to change physical activity levels at the baseline, midway and end-of-study motivation questionnaire, by study arm (D: diet; PA: physical activity; D+PA: diet and physical activity; C: control). The Diet, Physical Activity and Mammography trial, Florence, Italy, 2009–2012; Figure S3. Mean score to the question about confidence to be able to change eating habits at the baseline, midway and end-of-study motivation questionnaire, by study arm (D: diet; PA: physical activity; D+PA: diet and physical activity; C: control). The Diet, Physical Activity and Mammography trial, Florence, Italy, 2009–2012; Figure S4. Mean score to the question about confidence to be able to change physical activity levels at the baseline, midway and end-of-study motivation questionnaire, by study arm (D: diet; PA: physical activity; D+PA: diet and physical activity; C: control). The Diet, Physical Activity and Mammography trial, Florence, Italy, 2009–2012; Figure S5. Mean score to the question about willingness to participate in this type of research activities at the baseline, midway and end-of-study motivation questionnaire, by study arm (D: diet; PA: physical activity; D+PA: diet and physical activity; C: control). The Diet, Physical Activity and Mammography trial, Florence, Italy, 2009–2012.
